# Restoration of Strip Crown with a Resin-Bonded Composite Cement in Early Childhood Caries

**DOI:** 10.1155/2013/581934

**Published:** 2013-09-24

**Authors:** Mi-ae Jeong, Ah-hyeon Kim, Youn-soo Shim, So-youn An

**Affiliations:** ^1^Department of Dental Hygiene, Kangwon National University, Samcheok, Republic of Korea; ^2^Department of Preventive Dentistry and Public Oral Health, Research Center for Orofacial Hard Tissue Regeneration, College of Dentistry, Yonsei University, Seoul, Republic of Korea; ^3^Department of Dental Hygiene, Cheongju University, Cheongju, Republic of Korea; ^4^Pediatric Dentistry, College of Dentistry, Wonkwang University, Sanbon 435-040, Republic of Korea

## Abstract

*Background*. Early childhood caries is a widely prevalent disease throughout the world. It is necessary to treat this condition in early childhood; however, child behavior management may be particularly challenging during treatment. To overcome this challenge, we used Carigel to remove caries and RelyX Unicem resin cement for strip crown restoration. It not only has the desired aesthetic effect but is also more effective for primary teeth, which are used for a shorter period than permanent teeth are. *Case Presentation*. We report a case of three pediatric patients with early childhood caries, in whom caries was removed by using Carigel to avoid the risk of pulpal exposure associated with high-speed handpieces. Subsequently, aesthetic restoration was performed using strip crown with RelyX Unicem self-adhesive resin cement. *Conclusion*. RelyX Unicem has the following advantages: (1) not requiring have any special skills for the dentist for performing the procedure, (2) decreased occurrence of bubbles during injection of the cement, and (3) overall short duration of the procedure. Thus, it is appropriate for the treatment of pediatric patients whose behavior is difficult to manage. However, further studies are required in order to establish the use of RelyX Unicem as a stable restorative material in early childhood caries.

## 1. Introduction

Early childhood caries (ECC) commonly affects the maxillary anterior teeth and is characterized by the presence of caries, that is, one or more missing, decayed, or filled tooth surfaces on any primary tooth of children aged less than 71 months [1]. ECC can lead to loss of primary teeth. From the perspective of the health of permanent teeth, proper diet, and aesthetic appreciation, the importance of primary teeth cannot be ignored [2]. In addition, with the loss of primary teeth, children lose the ability to pronounce fricative and sibilant sounds, causing them to develop an inaccurate language pattern. Furthermore, ECC, when left untreated, results in the development of abscesses, pain, and malocclusion. As per national oral health investigation (2010), caries experience rate of primary tooth was 61.4% and average number of decayed teeth was 2.2 in Korean population. The high incidence of ECC also hinders education as it results in nonattendance in school. Moreover, the treatment costs involved becomes a great burden in a country promoting public health care [3]. Therefore, early discovery, diagnosis, and treatment of ECC are necessary.

A painless treatment of dental caries is important in the case of pediatric patients visiting a dentist for the first time. A high-speed handpiece is conventionally used to treat dental caries. This surgical method can prevent unwanted elimination of sound teeth; however, it involves the possibility of damaging the pulp, which may be exposed due to the generation of excessive heat [1, 4].

A new method for treating dental caries that involves no drill, running water, or electricity was presented at the headquarters of the World Health Organization on World Health Day in 1994. This new approach, termed “Atraumatic Restorative Treatment,” involves manual cleaning of the dental cavities with hand instruments and restoration of the cavities with an adhesive fluoride-releasing material [5]. In addition, chemomechanical techniques using rotary instruments are an alternative method to conventional caries removal techniques. 

**Figure 1:**
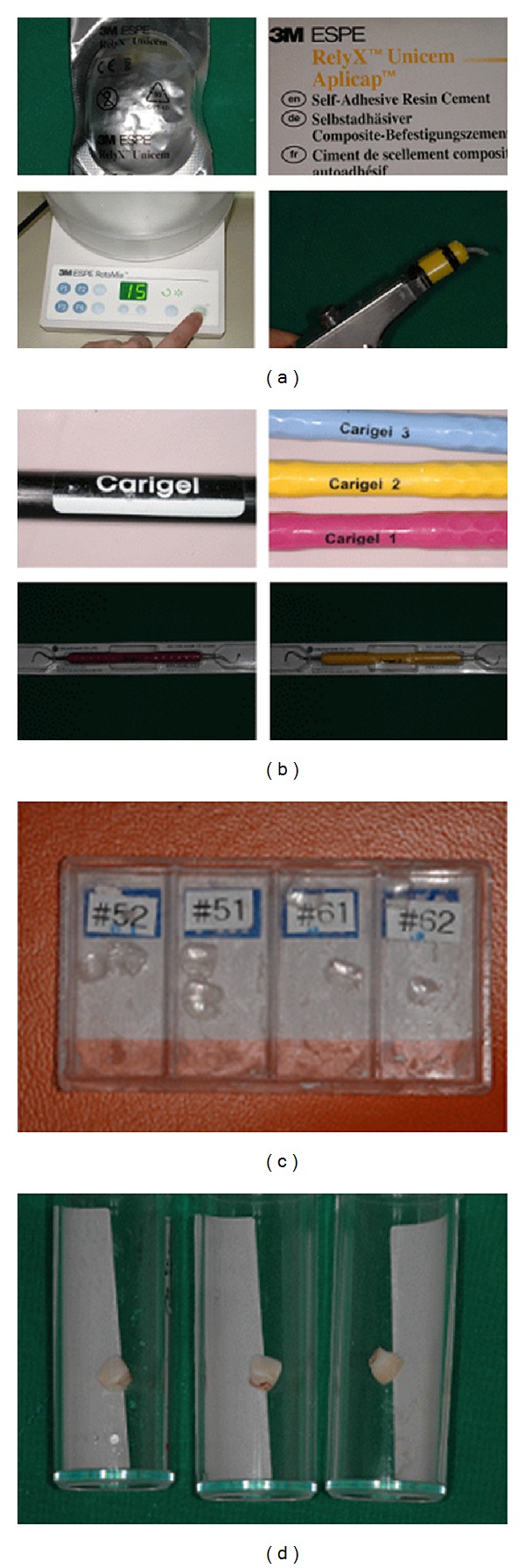
(a) RelyX Unicem. (b) Carigel. (c) Strip crown. (d) Primary tooth restored with RelyX Unicem. The patient used restoration until primary tooth is replaced by permanent tooth.

In the 1970s, Habib et al. [6] and Kronman et al. [7] studied the effect of a nonspecific proteolytic agent, sodium hypochlorite (NaOCl), on carious dentine. Unfortunately, NaOCl not only dissolved necrotic organic material but also affected sound dentine [8]. Further work led to the development of a new chemomechanical agent Carigel (Foryoudent, Busan, Korea., Figure 1(b)). It consists of a 2-component gel, supplied in 2 different syringes. The first syringe contains glutamic acid, leucine, lysine, sodium chloride, erythrosin, carboxymethylcellulose, water, and sodium hydroxide and has a pH value of 11. The second syringe contains 0.5% NaOCl and alanine aminotransferase. 

 Full coronal restoration of carious primary incisors is indicated in the following cases: (1) caries is present on multiple surfaces; (2) the incisal edge is involved; (3) extensive cervical decalcification is present; (4) pulpal therapy is indicated; (5) caries may be minor, but oral hygiene is very poor (high-risk patients); (6) the child's disruptive behavior makes placing Class II restorations difficult [9].

The restorative method generally used to treat ECC involves an open-faced stainless steel crown. When the aesthetic demand is high, restoration with a strip crown (3M ESPE, USA., Figure 1(c)) is restrictively performed. With respect to restoration, patients now seem to emphasize on improved aesthetics rather than function, especially in the case of anterior teeth. Using an open-faced stainless steel crown improves the aesthetics more than the stainless steel crown [10]. However, in Korea, the procedure that uses open-faced stainless steel crown has been used for a longer time than the strip crown because the handmade open-faced stainless steel crown is preferred over the commercial open-faced stainless steel crown (e.g., Nusmile, Chang crown). Usually anterior stainless steel crown (e.g., Unitek crowns-stainless steel primary anterior, 3M ESPE, USA, Kids crowns, SHINHUNG company, Korea) are used and they are cemented to the primary anterior tooth. After the cement is hardened, which could take several minutes, the labial surface of anterior stainless steel crown is cut exposing the tooth surface, following which resin is bonded to the tooth surface. However, the cement needs to be hardened before the resin is applied to smooth out the irregular surfaces. Metal is exposed to the marginal region. Thus, in several cases, it is difficult to satisfy patients and guardians who have high aesthetic demands [11].

Composite resin was conventionally used as the restorative material for celluloid strip crown in ECC. Composite resin was first developed by Bowen in the 1960s to strengthen epoxy resin as a filler; since then, several advancements have been made to enhance its physical properties such as its resistance to abrasion, polymerization shrinkage, and bonding strength of dentin. In addition, it is being used extensively to restore anterior teeth for improved aesthetics and to restore posterior teeth without applying too much pressure. However, composite resin is sensitive to the individual skills of a dentist and the process involved is considerably long; therefore, it is not convenient for use in pediatric patients, where behavioral management is relatively difficult compared to adults.

Here, we report a case of three pediatric patients with early childhood caries, in whom caries was removed by using Carigel; restoration was performed using a resin-bonded composite cement RelyX Unicem (3M ESPE Dental Products, 2 USA, Figure 1(a)).

## 2. Case Presentation 1

**Figure 2:**
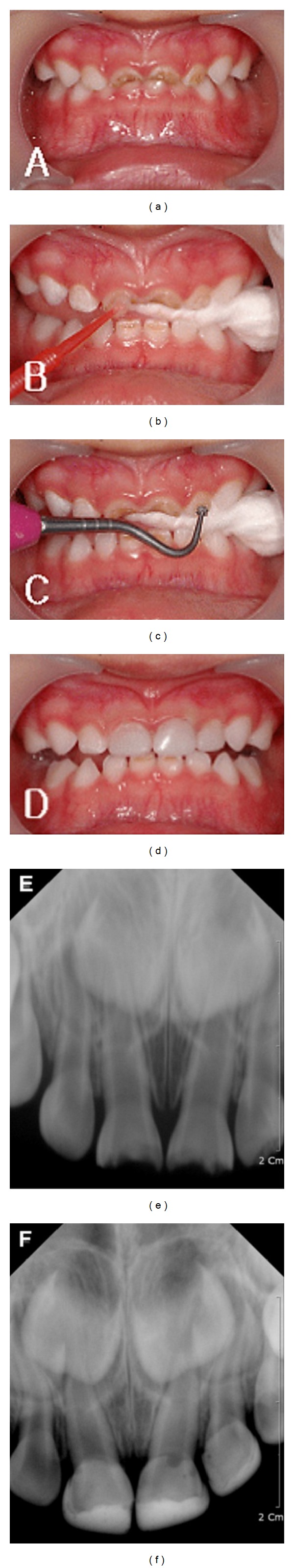
(a) Oral status of the patient before treatment, (b) application of Carigel, (c) removal of dental caries, (d) restoration with RelyX Unicem, (e) radiograph of the patient before treatment, and (f) radiograph of the patient treatment after 8 month.

In February 2010, an 18-month-old Korean girl presented with ECC at the Wonkwang University Dental Hospital (Figure 2(a)). She harbored a fear for dental treatment and was treated using Carigel (FORYOUDENT, Busan, Korea) to avoid the risk of pulp exposure when using a high-speed hand-piece. 

According to the manufacturer's instructions, the 2 syringes in which the gel is supplied were maintained at room temperature for approximately 1 hour before treatment [4]. Carigel was applied to the affected area by using a microbrush (Figure 2(b)). After 30 seconds, the gel was absorbed by using hand instrument 1, exclusively designed for Carigel. Caries was removed by using dedicated hand instruments 2 and #3. The process of applying the gel and removing caries by using hand instruments was repeated twice (Figure 2(c)). After the process of removing caries was completed, a 3-way syringe was used to wash the area to prevent the removed caries residue and gel from remaining on the tooth surfaces. A celluloid crown (3M ESPE, USA) was selected according to the size of the affected tooth after washing, and its shape was modified by clamping after cutting it to the appropriate length. The RelyX Unicem (3M ESPE, USA) was mixed with an automixer and then injected into the crown form followed by an expert surgeon applying the crown form to the affected tooth, which was then removed after photopolymerization (Figure 1(d)).

## 3. Case Presentation 2

**Figure 3:**
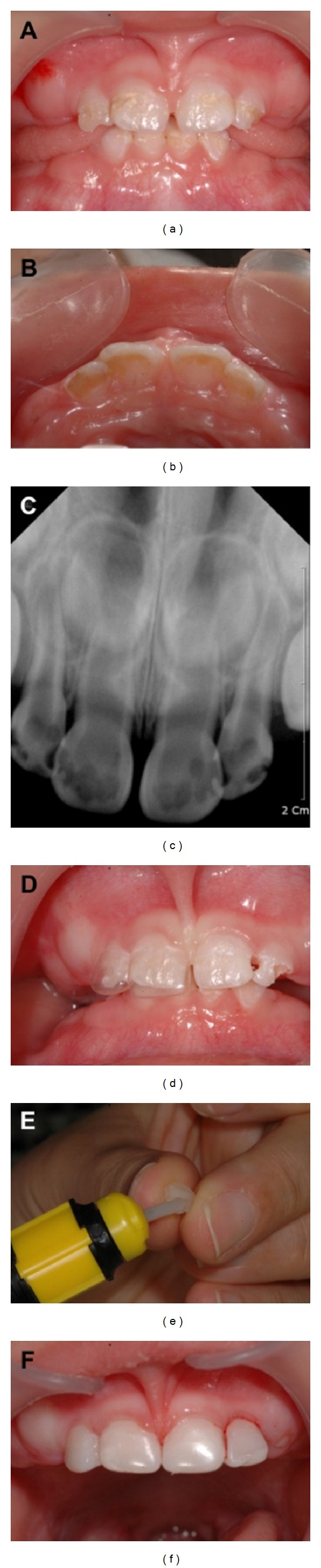
(a) Oral status of the patient before treatment (labial surface), (b) oral status of the patient before treatment (lingual surface), (c) radiograph of the patient before treatment, (d) mounting of strip crown on patient's tooth, (e) filling strip crown with RelyX Unicem, and (f) restoration with RelyX Unicem.

In August 2009, a 16-month-old Korean girl presented with ECC at the Wonkwang University Dental Hospital (Figures 3(a) and 3(b)). Caries did not invade the pulp, but was very close to the pulp (Figure 3(b)). She was treated using Carigel (Foryoudent, Busan, Korea) to avoid the risk of pulp exposure while using a high-speed hand-piece. After the process of removing caries was completed, a 3-way syringe was used to wash the area to prevent the removed caries residue and gel from remaining on the surface of the tooth. A celluloid crown (3M ESPE, USA) was selected according to the size of the affected tooth after washing, and its shape was modified by clamping after cutting it to the appropriate length (Figure 3(d)). RelyX Unicem (3M ESPE, USA) was mixed with an automixer and then injected into the crown form (Figure 3(e)) followed by application of the crown form to the affected tooth, which was then removed after photopolymerization (Figure 3(f)).

## 4. Case Presentation 3

**Figure 4:**
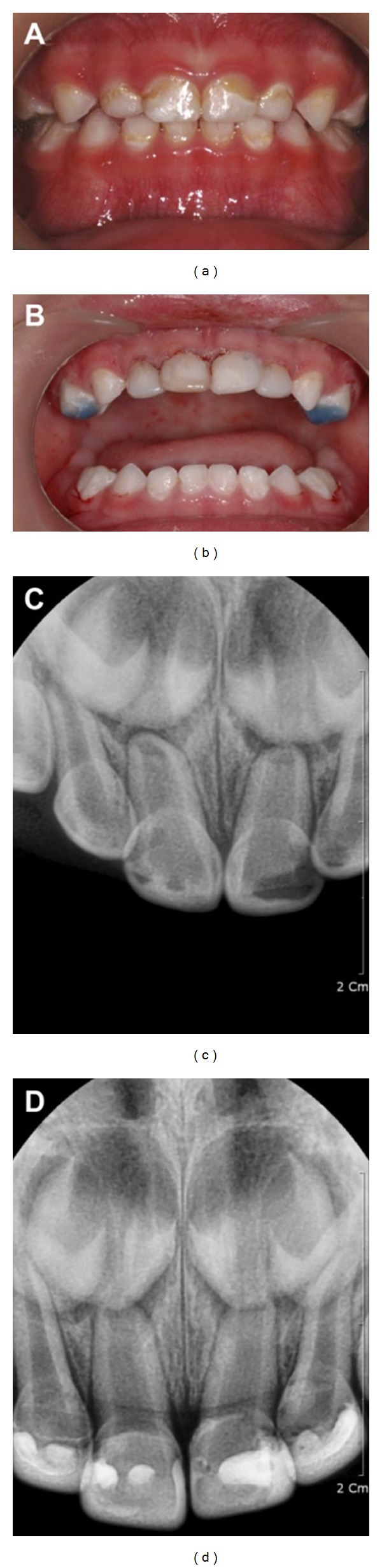
(a) Oral status of the patient before treatment and (b) restoration with RelyX Unicem. In order to avoid anterior teeth biting, 54 and 64 teeth were restored with blue resin. (c) Radiograph of the patient before treatment and (d) radiograph of the patient after treatment.

In May 2013, a 22-month-old Korean girl presented with ECC at the Wonkwang University Dental Hospital (Figures 4(a) and 4(c)). She was treated using Carigel (Foryoudent, Busan, Korea) to avoid the risk of pulp exposure while using a high-speed hand-piece. She was treated using Carigel and RelyX Unicem (3M ESPE, USA) in the same way as case 2. In order to avoid anterior teeth biting, 54 and 64 teeth were restored with blue resin (Figure 4(b)).

To meet the rising aesthetic needs, resin-bonded composite strip crowns are the preferred choice of many clinicians, mainly because of the superior aesthetics and the ease of repair in the event that the crown chips or fractures [12]. Restoration of the primary incisors with single bond and Z100 using strip crowns (all by 3M ESPE Dental Products, Seefeld, Germany) is popularly performed in some countries. At our hospital, we also generally used the packable resin Z100 (P shade) as the restorative material for filling the strip crown. In this case, the strip crown could be selected and RelyX Unicem (3M ESPE Dental Products, USA) was used instead of Z100. RelyX Unicem is a unique resin cement. The flexural strength of RelyX Unicem is 63 MPa, which is lower than that of Z100, a packable resin, whose flexural strength is 160 MPa. However, considering that restored primary teeth are used for a shorter period than permanent teeth, RelyX Unicem has the following merits: (1) the dentists performing the procedure do not require any special skills, (2) it facilitates decreased occurrence of bubbles during injection of the material, and (3) it involves a relatively shorter time. Therefore, it is more appropriate for the treatment of caries in pediatric patients, in whom behavioral management may be difficult. 

Here, we have reported one such case in which RelyX Unicem was used for the restoration in ECC. We applied this method to a number of children. The majority of patients used restoration until restored deciduous tooth was replaced by permanent tooth (Figure 1(d)). However, to stably use RelyX Unicem as a generally accepted restorative material for primary teeth in pediatric patients with ECC, it is important to compare its physical properties such as microstrength and polymerization shrinkage to those of other restorative materials, which have been traditionally used in laboratories. Further studies must be performed to evaluate the long-term prognosis of ECC treated with Carigel and strip crowns using RelyX Unicem.
